# Chemical Composition and Quality Assessment of *Apis mellifera* Honey in Australia

**DOI:** 10.1002/fsn3.70160

**Published:** 2025-04-15

**Authors:** Jamie Ayton, Leanne Groves, Jessica Berry

**Affiliations:** ^1^ NSW Department of Primary Industries Wagga Wagga New South Wales Australia; ^2^ Hive and Wellness PTY LTD Brisbane Queensland Australia

**Keywords:** Australia, chemical composition, diastase, free acidity, HMF, honey, moisture content, quality, sugar

## Abstract

Honey is a natural product highly valued for its nutritional, therapeutic, and economic significance, with its quality and composition influenced by botanical and geographical origins. Australian honey is particularly unique due to its indigenous floral sources and diverse geographic regions. In this study, 267 honey samples were collected from across Australia during the 2020–2023 production seasons. Physicochemical properties, including moisture content (regional means ranged from 15.4 to 17.8 g/100 g), pH (3.97–4.45), free acidity (13.0–26.8 meq/kg), diastase activity (17.4–39.5 DN, Schade scale) 5‐hydroxymethylfurfural (HMF) (2.1–15.7 mg/kg), electrical conductivity (0.37–0.88 mS/cm), water‐insoluble solids, and sugar composition, were analyzed. Significant correlations were observed between electrical conductivity and pH (*r* = 0.64) as well as HMF and free acidity (*r* = 0.50). The results demonstrate a wide diversity in the composition of Australian honey, influenced by climatic conditions, flowering periods, and postharvest practices. These findings provide valuable insights into the quality and uniqueness of Australian honey, paving the way for further research to expand the database, identify distinct characteristics, and ensure product authenticity and integrity.

## Graphical abstract

In this study, 267 honey samples were collected from across Australia during the 2020–2023 production seasons. Physicochemical properties, including moisture content, pH, free acidity, diastase activity, 5‐hydroxymethylfurfural (HMF), electrical conductivity, water‐insoluble solids, and sugar composition, were analyzed. The results demonstrate a wide diversity in the composition of Australian honey, influenced by climatic conditions, flowering periods, and postharvest practices. These findings provide valuable insights into the quality and uniqueness of Australian honey.

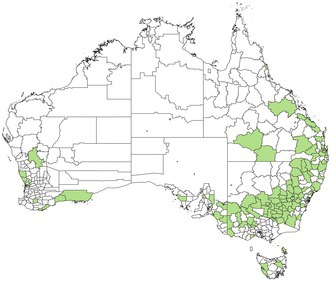

## 1 Introduction

Honey is a complex natural product consisting mainly of sugars and water as well as other minor components including proteins, pigments, enzymes, antioxidants, and volatile compounds (Al et al. 2009; Yao et al. 2003). The assessment of honey quality is dependent on numerous factors, including both the physical and chemical attributes detailed in this study.

The Australian honey market presents several opportunities for producers and industry stakeholders. As consumers increasingly prioritize healthy dietary choices, there is a growing demand for natural, nutritious products. Honey, being a natural sweetener, is increasingly favored overprocessed sugars for its perceived health benefits. While some honey is marketed based on anecdotal evidence of distinctive traits, such as specific floral sources or health claims, there is potential to define these traits using scientifically derived data, which can be used when marketing the product. The distinctive traits of Australian honey are particularly appealing to international consumers, presenting opportunities for marketing and export to those regions.

Australia's diverse native flora, including over 700 species of eucalyptus, significantly influences honey composition (Sniderman et al. 2018; Somerville 2019). The distinct biochemical profiles of nectar from different plant species impart unique traits to the honey derived from those species, such as variations in sugar composition and moisture content. For instance, nectar from some eucalyptus species results in honey with lower moisture concentration due to its concentration (Bogdanov et al. 2004) while sugar composition is dependent on the floral source of the honey (da Silva et al. 2016). The diverse Australian landscape, which includes arid inland regions, coastal areas, and tropical zones, significantly influences honey composition and physiochemical properties. This includes the presence and abundance of floral sources in these areas as well as the effects of climatic conditions during the honey ripening process. Understanding the physiochemical properties of Australian honey is critical. It allows for the development of reference databases that can be used to establish quality benchmarks and identify adulteration.

Australian beekeepers are estimated to produce around 37,000 tons of honey each year, with the European honey bee, *Apis mellifera* , being the primary species responsible for this production. In 2019, the Australian Bureau of Agricultural Resource Economics and Sciences (ABARES) estimated the honey and beeswax sales value at $147 million. Honey accounts for around 80% of the value of Australian beekeeping products, making it the primary revenue source for beekeepers (Clarke and LeFeuvre 2021). The Australian honey industry represents around 2% of the global honey production of approximately 1.9 million tonnes (Sharma et al. 2023). However, focusing on producing a high‐quality product, rather than high volumes, could be advantageous for Australian honey producers. To accomplish this, determining the composition of Australian honey using well‐established, standardized analytical methods is necessary. Previous studies have characterized some of the parameters discussed in this report; however, these have often relied on samples from limited geographical areas or had a small sample size. As beekeeping and honey production occur across all Australian states and territories, a comprehensive data set is required to accurately characterize the composition of Australian honey. This includes considering different geographic areas with their unique indigenous flora as well as variations in beekeeping and processing practices and seasonal influences. The present study aims to characterize the physiochemical properties and quality of honeys from various regions across Australia over three seasons.

## 2 Materials and Methods

### 2.1 Honey Samples

**FIGURE 1:**
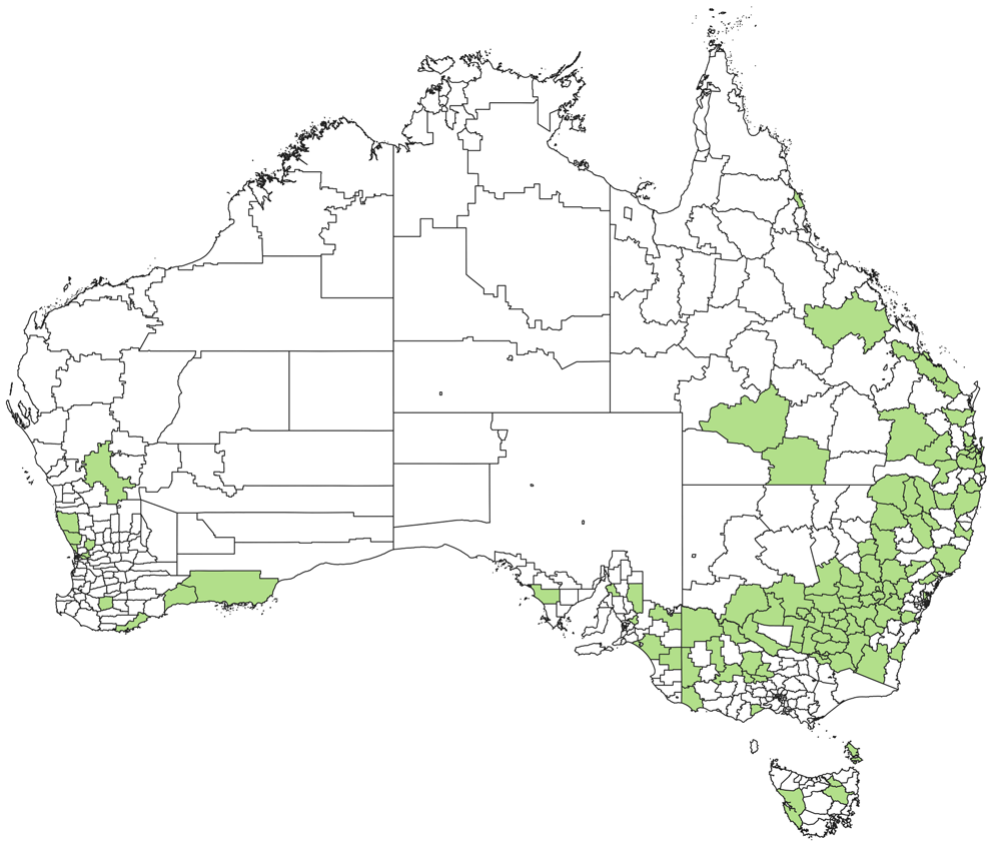
Map of Australia showing honey collection sites.

This study involved the collection of 267 honey samples from commercial beehives in various regions across six Australian states between 2021 and 2023. The samples were collected from local beekeepers and honey packers. The collection encompassed samples from New South Wales (NSW), Australia's largest honey‐producing state with 115 samples. Additionally, samples were obtained from Queensland (Qld, 49 samples), South Australia (SA, 44 samples), Victoria (Vic, 25 samples), Tasmania (Tas, 9 samples), and Western Australia (WA, 25 samples) (Figure 1). Beekeepers and honey packers provided details such as hive location and processing conditions. To safeguard the confidentiality and privacy of beekeepers', sample categorization was conducted based on the state of production. Following extraction, samples were securely transported to the laboratory in sealed plastic containers at ambient temperature and stored at 4°C until subjected to analysis.

### 2.2 Physiochemical Parameters

Moisture content was determined using a refractometer (ATAGO Abbe Refractometer) at 20°C, following the International Honey Commission method 1 “Determination of moisture, refractometric method” (IHC 1997).

Free acidity and pH were determined using a pH meter (SmartChem Lab), following the International Honey Commission method 4 “pH and free acidity.” For pH measurement a solution was prepared by dissolving 10 g honey in 75 mL of deionized water. Free acidity was determined through titration with 0.1 M NaOH until a pH of 8.30 was reached (IHC 1997).

Diastase activity was determined using International Honey Commission method 6.2 “Determination of diastase activity with Phadebas.” A buffered solution of soluble starch with dye and honey was incubated at 40°C, and the absorbance was measured at 620 nm on a spectrophotometer (Thermo Scientific) (IHC 1997).

The 5‐hydroxymethylfurfural (HMF) content of the honey samples was determined following the International Honey Commission method 5.1 “Determination of hydroxymethylfurfural by HPLC,” following the guidelines in the White method (IHC method 5.2 “Determination of hydroxymethylfurfural after White”) (IHC 1997). Each sample solution consisted of 5 g honey, with Carrez reagents (I and II) added and deionized water used to make up to a total volume of 50 mL. Subsequently, a HMF standard solution was prepared and diluted into various concentrations ranging from 0 to 10 mg/L to establish an external calibration curve. Chromatographic analysis was conducted by reverse phase high performance liquid chromatography (RP‐HPLC) using an Agilent 1260 Infinity HPLC equipped with a photodiode array detector (PDA) set at 285 nm. A ThermoScientific Hypersil ODS column (125 × 4 mm, 5 μm particle size) was used, with a mobile phase composed of 90:10 deionized water: methanol, with a flow rate of 0.8 mL/min and sample injection volume of 20 μL. Analysis and data acquisition were done using OpenLAB Chemstation version 3.2. The HMF content of the sample was determined by establishing a linear regression of the peak areas of the sample against those of the standard solutions.

Electrical conductivity was determined using International Honey Commission method 2 “Determination of electrical conductivity.” A solution was prepared by dissolving the equivalent of 10 g dry weight of honey (adjusted based on the previously determined moisture content) in 50 mL deionized water. The electrical conductivity of the solution was then measured using a conductivity meter with a temperature probe (SmartChem Lab). To ensure consistency, a correction factor was applied, as described in the method, to standardize the results to 20°C (IHC 1997).

Water insoluble solids were determined using International Honey Commission method 8 “Determination of insoluble matter.” Honey (20 g) was filtered through preweighed sintered glass crucibles (G3, pore size 15–40 mm) using 200 mL deionized water heated to 80°C. This procedure was repeated five times. Subsequently, the crucible was dried at 135°C for 1 h, allowed to cool in a desiccator and then reweighed. The sample was then returned to the oven for 30‐min intervals until constant weight was achieved (IHC 1997).

The sugar composition of each honey sample was determined using International Honey Commission method 7.2 “Determination of sugars by HPLC.” Honey (5 g) was dissolved in 40 mL of deionized water, then transferred to a 100 mL flask containing 25 mL methanol, and made up to 100 mL with deionized water. Similarly, a standard solution was prepared by dissolving fructose, glucose, and sucrose in the same manner as the samples (IHC 1997). Chromatographic analysis was conducted using reverse phase high performance liquid chromatography (RP‐HPLC). An Agilent 1290 Infinity HPLC with a refractive index detector (RID) was used. A ThermoScientific Hypersil Gold Amino column (150 × 4.6 mm, 3 mm particle size) was used, with a mobile phase composed of 85:15 acetonitrile:water. The mobile phase flow rate was 0.9 mL/min for 8.5 min, which was increased to 1.1 mL/min after 9 min and decreased to 0.9 mL/min after 20 min. The sample injection volume was set at 20 μL, the column temperature at 35°C and the RID temperature at 40°C. Analysis and data acquisition were performed using OpenLAB Chemstation version 3.2. Identification and quantification of honey sugars were performed by comparing retention times with those of standard sugars.

The limit of quantification (LOQ) was calculated for all analytical methods (Table 1). Results below the LOQ were reported as < LOQ.

**TABLE 1:** Physiochemical parameters of honey samples.

Region	Season	Moisture (g/100 g)	pH	Free acidity (mEq/kg)	Diastase (DN, Schade scale)	HMF (mg/kg)	EC (mS/cm)	WIS (g/100 g)
NSW	2020/21 (*n* = 30)	16.6 ± 1.0^abc^	3.97 ± 0.23^a^	18.7 ± 5.0^abc^	22.8 ± 8.0^ab^	4.5 ± 5.2^ab^	0.37 ± 0.21^a^	0.06 ± 0.09^a^
	2021/22 (*n* = 67)	16.6 ± 1.1^abc^	4.09 ± 0.31^abc^	18.4 ± 6.3^ab^	24.6 ± 9.2^abc^	5.8 ± 7.2^ab^	0.47 ± 0.26^ab^	0.06 ± 0.08^a^
	2022/23 (*n* = 18)	17.2 ± 1.3^c^	4.13 ± 0.31^abc^	20.5 ± 6.8^abc^	21.9 ± 9.7^ab^	6.9 ± 7.9^ab^	0.54 ± 0.22^ab^	0.07 ± 0.08^a^
Qld	2020/21 (*n* = 11)	17.3 ± 1.2^c^	4.23 ± 0.28^abc^	23.3 ± 14.3^bc^	17.5 ± 7.2^a^	15.7 ± 28.6^b^	0.65 ± 0.22^abc^	0.03 ± 0.03^a^
	2021/22 (*n* = 30)	17.4 ± 0.8^c^	4.14 ± 0.20^abc^	21.0 ± 3.8^abc^	17.8 ± 3.9^a^	15.1 ± 13.2^b^	0.55 ± 0.20^abc^	0.04 ± 0.05^a^
	2022/23 (*n* = 8)	16.8 ± 1.0^abc^	4.09 ± 0.22^abc^	17.7 ± 5.2^ab^	20.3 ± 5.6^ab^	6.8 ± 5.3^ab^	0.44 ± 0.09^ab^	0.26 ± 0.33^b^
SA	2020/21 (*n* = 8)	15.4 ± 0.4^a^	4.38 ± 0.13^bc^	15.2 ± 4.2^ab^	17.4 ± 4.6^a^	3.8 ± 4.8^ab^	0.78 ± 0.19^bc^	0.12 ± 0.05^ab^
	2021/22 (*n* = 9)	17.8 ± 2.3^c^	4.41 ± 0.57^bc^	13.7 ± 6.4^a^	17.5 ± 5.4^a^	3.4 ± 3.5^ab^	0.55 ± 0.29^abc^	0.19 ± 0.15^ab^
	2022/23 (*n* = 27)	17.1 ± 1.0^bc^	4.37 ± 0.36^abc^	13.0 ± 2.2^a^	23.0 ± 5.8^ab^	2.1 ± 1.5^a^	0.51 ± 0.23^ab^	0.12 ± 0.07^ab^
Vic	2020/21 (*n* = 0)	—	—	—	—	—	—	—
	2021/22 (*n* = 10)	15.6 ± 1.0^a^	4.32 ± 0.24^abc^	19.1 ± 7.0^abc^	31.3 ± 0.7^bcd^	4.1 ± 3.4^ab^	0.65 ± 0.24^abc^	0.06 ± 0.06^a^
	2022/23 (*n* = 15)	17.6 ± 0.7^c^	4.23 ± 0.15^abc^	19.3 ± 3.3^abc^	26.7 ± 0.6^abc^	3.6 ± 4.3^ab^	0.56 ± 0.20^abc^	0.12 ± 0.07^ab^
Tas	2020/21 (*n* = 4)	15.7 ± 0.5^ab^	4.02 ± 0.25^ab^	23.1 ± 0.7^bc^	34.4 ± 13.0^cd^	3.9 ± 3.9^ab^	0.47 ± 0.15^ab^	0.09 ± 0.09^ab^
	2021/22 (*n* = 5)	15.7 ± 1.9^ab^	4.34 ± 0.22^abc^	26.8 ± 7.9^c^	39.5 ± 10.6^d^	3.2 ± 1.9^ab^	0.70 ± 0.19^abc^	0.06 ± 0.05^a^
	2022/23 (*n* = 0)	—	—	—	—	—	—	—
WA	2020/21 (*n* = 0)	—	—	—	—	—	—	—
	2021/22 (*n* = 0)	—	—	—	—	—	—	—
	2022/23 (*n* = 25)	16.4 ± 1.0^ab^	4.45 ± 0.24^c^	19.7 ± 4.8^abc^	25.1 ± 12.3^abc^	3.2 ± 2.9^ab^	0.88 ± 0.34^c^	0.26 ± 0.32^b^
Range		13.6–22.4	3.51–5.52	7.0–64.6	< LOQ—63.6	< LOQ—95.2	0.15–1.91	< LOQ—1.20
Limit of quantification		0.5	0.46	1.0	0.8	1.0	0.05	0.02
CODEX limit		< 20	—	< 50	> 8	< 40	< 0.8	< 0.1

*Note:* Results are mean ± SD. Values followed by the same letter in the column do not differ significantly by Tukey's test (*p* < 0.05).

Abbreviations: EC, electrical conductivity; HMF, hydroxymethylfurfural; NSW, New South Wales; Qld, Queensland; SA, South Australia; Tas, Tasmania; Vic, Victoria; WA, Western Australia; WIS, water insoluble solids.

Data analysis was performed using XLSTAT (version 2022.5.1). Subsequently, the data were exported to MetaboAnalyst 5.0 software (https://www.metaboanalyst.ca/) for principal component and partial least squares discriminant analysis.

## 3 Results and Discussion

### 3.1 Physiochemical Parameters

The physiochemical properties of the honey samples analyzed are presented in Table 1.

The moisture for the honey samples studied ranged from 13.6 to 22.4 g/100 g. The lowest average moisture content was 15.4 g/100 g in honey samples from SA during the 2020/2021 season, whereas the highest was 17.8 g/100 g in honey samples from SA during the 2021/2022 season (Table 1). Two samples from SA from the 2021/2022 season exceeded the Codex moisture limit of 20 g/100 g (CODEX 2001). Both originated from the same beekeeper, shared similar floral sources and were extracted at the same time, suggesting these factors influenced moisture content. Various factors such as seasonal variations, floral sources, climatic conditions, harvesting processes, honey maturity, and storage conditions can influence moisture content in honey, contributing to the considerable range shown in this study. The moisture content ranges observed in Australian honey are similar to findings in other studies, both domestic (Ajlouni and Sujirapinyokul 2010) and internationally (da Silva et al. 2016).

Honey's natural acidity, primarily due to gluconic acid, plays a key role in microbial inhibition (Majewska et al. 2019). There is no set limit or range for pH in honey. Prior research has documented pH levels ranging between 3.2 and 5.6 (Karabagias et al. 2014a; Knİghts et al. 2023). In this study, the pH values ranged from 3.51 to 5.52, with the highest mean (4.45) observed in WA during the 2022/2023 season and the lowest mean (3.97) in NSW during the 2020/2021 season (Table 1). The presence of natural organic acids and other components, such as esters, lactones, and inorganic ions, influences the free acidity of honey. Factors such as geographical origin and harvest season can affect free acidity (da Silva et al. 2016; Tornuk et al. 2013). However, a high concentration of free acidity may suggest honey fermentation due to the presence of yeast, as the alcohol, produced during fermentation, is converted into organic acids (Ajlouni and Sujirapinyokul 2010). In this study, free acidity ranged from 7.0 to 64.6 mEq/kg, with one sample exceeding the Codex limit (< 50 mEq/kg) (CODEX 2001). This sample was from a tropical region in northern Australia, where climatic conditions likely influenced the honey's composition. The highest mean free acidity was 26.8 mEq/kg during the 2021/2022 season in Tasmania, whereas the lowest mean was 13.0 mEq/kg in South Australia during 2022/2023 (Table 1). The free acidity values detailed in this report are similar to those documented in other studies (Devillers et al. 2004; Laaroussi et al. 2020).

Diastase, an enzyme naturally present in honey, originates from secretions of bees' hypopharyngeal glands, with a minor amount coming from the nectar of plants (Ohashi et al. 1999). The activity of this enzyme decreases as the honey ages or with prolonged exposure to heat, making it a useful indicator of freshness or potential overheating during processing (Nagai et al. 2009). The Codex limit for diastase activity is > 8 DN (Schade scale) (CODEX 2001). In this study, the diastase activity ranged from < LOQ to 63.6 DN, with two samples lower than the international limit. Both samples were from Western Australia during the 2022/2023 season. There were no common descriptions between the samples, although the geographic locations where the honey was produced were neighboring areas. The lowest mean diastase activities were in Qld and SA during the 2020/2021 and 2021/2022 seasons (17.4–17.8 DN, respectively), whereas the highest means were in Tas (34.4 and 39.5) during the 2020/2021 and 2021/2022 seasons, respectively (Table 1). Diastase activity varied within each state, similar to results from other studies (Bentabol Manzanares et al. 2014; Horčinová Sedláčková et al. 2022).

5‐Hydroxymethylfurfural (HMF) is formed in honey through the Maillard reaction or as a by‐product of the dehydration of sugar and is generally present in low concentrations in fresh foods. However, overheating or high ultraviolet (UV) radiation exposure during extraction, processing, and/or storage can elevate HMF levels in honey (Zappalà et al. 2005). The Codex limit for HMF in honey is < 40 mg/kg (CODEX 2001). Three samples exceeded 40 mg/kg, one (40.6 mg/kg) from southern New South Wales, with the others (74.7 and 95.2 mg/kg) from central and northern Queensland, likely influenced by the tropical climate of that region. Processing, storage, and packaging can elevate HMF concentrations. However, the samples for this study were raw, unfiltered, and stored in cool conditions immediately after collection, minimizing these effects. Conversely, 12.5% of the samples showed HMF concentrations below the limit of quantification. Most states showed relatively low mean HMF concentrations (ranging from 2.1 to 6.9 mg/kg), whereas mean values from Qld during the 2020/2021 and 2021/2022 seasons were notably higher (15.7 and 15.1 mg/kg, respectively), attributable to the tropical climate previously mentioned (Table 1). HMF values in this study align with previous findings (da Silva et al. 2016). The comparatively low mean HMF concentrations in Australian honey reflect the industry's adherence to sound practices in hive management, honey extraction, processing, and storing and may serve as a basis for marketing strategies.

The electrical conductivity (EC) of honey is influenced by its mineral and organic acid content, with higher concentrations resulting in elevated conductivity values (Ratiu et al. 2020). While some researchers suggest that EC varies depending on the floral origin of the honey (Dobrinas et al. 2022), others have found no such correlation (Kaškonienė et al. 2010). The codex limit for EC is < 0.8 mS/cm (CODEX 2001). In this study, EC ranged from 0.15 to 1.91 mS/cm, with approximately 15% of samples exceeding the Codex limit. The mean EC exceeded the Codex limit in Western Australia during 2022/2023 (0.88 mS/cm), yet mean values for other states were below the Codex limit, ranging from 0.37 mS/cm in New South Wales during 2020/2021 to 0.78 mS/cm in South Australia during 2020/2021 (Table 1). The mean values are similar to those reported in other studies (Boussaid et al. 2018; Chakir et al. 2016). The samples analyzed in this project were not filtered by beekeepers or prior to laboratory analysis. Therefore, the presence of pollen, dust, and inorganic matter in the samples likely contributed to the results.

Water insoluble solids (WIS) content in honey originate from organic and inorganic sources, including components such as beeswax, bee remnants, pollen, and dust. The clarity of honey is an important criterion for consumers, prompting beekeepers to use techniques such as centrifugation, filtration, and settling procedures during storage to remove these components (Albu et al. 2021). However, the clarification process may inadvertently alter some of the characteristics of the end product, such as the removal of pollen or changes in HMF or diastase content if heat is applied. The international limit for WIS is < 0.1 g/100 g (CODEX 2001). Results ranged from < LOQ to 1.20 g/100 g, with approximately 25% of samples exceeding the Codex limit. Mean WIS results varied between states and seasons, with the lowest values in Queensland during the 2020/2021 and 2021/2022 seasons (0.03 and 0.04 g/100 g, respectively), and the highest in Queensland and Western Australia during the 2022/2023 season (both 0.26 g/100 g). The mean WIS value exceeded the Codex limit in each season in South Australia and in the 2022/2023 season in Victoria (Table 1). No discernible patterns were observed regarding floral sources or beekeepers for honey samples that exceeded the Codex limit. The mean values for WIS shown in this study are comparable to those shown for other studies which used the same type of sample (raw, unfiltered samples directly from beekeepers) (Albu et al. 2021; Santos et al. 2014).

Honey primarily consists of sugars, making up approximately 95% of the dry weight. When sucrose, the primary sugar in nectar, undergoes enzymatic hydrolysis, it yields fructose and glucose. However, other sugars are present in small quantities in honey, including sucrose, turanose, maltose, and trehalose (de la Fuente et al. 2011; Persano Oddo and Piro 2004).

In this study, the sum of fructose + glucose ranged from 62.6 to 78.6 g/100 g, meaning all samples were above the Codex limit of > 60 g/100 g (CODEX 2001). Mean results were lowest in Queensland during the 2021/2022 season (68.1 g/100 g), whereas the highest mean result was 74.1 g/100 g during the 2020/2021 season in South Australia (Table 2). Other studies have shown similar ranges for the sum of fructose + glucose to those in this study (Kahraman et al. 2010; Ouchemoukh et al. 2007).

**TABLE 2:** Characteristics of sugar in honey samples.

Region	Season	Sum Fructose + Glucose (g/100 g)	Fructose/Glucose ratio	Sucrose (g/100 g)
NSW	2020/21 (*n* = 30)	71.2 ± 2.0^abc^	1.32 ± 0.13^a^	0.9 ± 1.8^a^
	2021/22 (*n* = 67)	70.0 ± 1.9^ab^	1.28 ± 0.12^a^	0.6 ± 0.8^a^
	2022/23 (*n* = 18)	71.1 ± 2.4^abc^	1.27 ± 0.13^a^	0.7 ± 0.8^a^
Qld	2020/21 (*n* = 11)	71.3 ± 4.1^abc^	1.27 ± 0.08^a^	< LOQ
	2021/22 (*n* = 30)	68.1 ± 2.0^a^	1.29 ± 0.14^a^	< LOQ
	2022/23 (*n* = 8)	72.0 ± 2.2^bc^	1.21 ± 0.09^a^	0.6 ± 0.8^a^
SA	2020/21 (*n* = 8)	74.1 ± 3.0^c^	1.24 ± 0.15^a^	< LOQ
	2021/22 (*n* = 9)	69.9 ± 3.2^ab^	1.35 ± 0.20^a^	< LOQ
	2022/23 (*n* = 27)	70.7 ± 3.3^abc^	1.28 ± 0.20^a^	0.8 ± 1.0^a^
Vic	2020/21 (*n* = 0)	—	—	—
	2021/22 (*n* = 10)	70.6 ± 1.6^abc^	1.38 ± 0.17^a^	0.6 ± 1.7^a^
	2022/23 (*n* = 15)	70.7 ± 3.8^abc^	1.32 ± 0.22^a^	< LOQ
Tas	2020/21 (*n* = 4)	73.2 ± 1.5^bc^	1.17 ± 0.08^a^	< LOQ
	2021/22 (*n* = 5)	71.0 ± 2.1^abc^	1.34 ± 0.32^a^	< LOQ
	2022/23 (*n* = 0)	—	—	—
WA	2020/21 (*n* = 0)	—	—	—
	2021/22 (*n* = 0)	—	—	—
	2022/23 (*n* = 25)	70.0 ± 2.9^ab^	1.28 ± 0.20^a^	< LOQ
Range		62.6–78.6	0.85–2.03	< LOQ—8.8
Limit of quantification (LOQ)		5.8	—	0.5
CODEX limit		> 60		< 5

*Note:* Results are mean ± SD. Values followed by the same letter in the column do not differ significantly by Tukey's test (*p* < 0.05).

Abbreviations: NSW, New South Wales; Qld, Queensland; SA, South Australia; Tas, Tasmania; Vic, Victoria; WA, Western Australia.

The fructose:glucose ratio (F:G) is sometimes used to evaluate physical characteristics of honey. Various sources indicate that levels below 1.3:1 (Escuredo et al. 2014) or 1.2:1 (da Silva et al. 2016) may accelerate crystallization in honey due to glucose's lower solubility in water compared to fructose. The F:G in this study ranged from 0.85 to 2.03. For those individual samples that showed relatively low or high F:G, floral source likely had the largest effect; however, a more targeted study would be needed to determine specific ranges for individual floral sources. Mean results from all regions were relatively close, ranging from 1.17 to 1.38. Other studies have reported similar ranges (El Sohaimy et al. 2015; Juan‐Borrás et al. 2014).

The Codex limit for sucrose is < 5 g/100 g (CODEX 2001). Sucrose content in honey can indicate intentional adulteration with sweeteners or unintentional adulteration caused by supplementary feeding sucrose solutions to ensure colonies survive during poor foraging conditions. However, authentic honey can sometimes have elevated sucrose levels due to premature harvesting, as the enzymatic transformation of sucrose into fructose and glucose may remain incomplete (Pita‐Calvo et al. 2017). In this study, sucrose ranged from < LOQ to 8.8 g/100 g. Nearly 70% of the samples from this study were below the limit of quantification for this method. Two samples exceeded the Codex limit for sucrose: one from New South Wales during the 2020/2021 season (8.8 g/100 g) which was noted to be from bees fed with sugar supplements, and another from Victoria during the 2021/2022 season (5.4 g/100 g). There were no indications from the beekeeper as to what may have contributed to the elevated sucrose levels in the Victorian sample. Mean results from each region show sucrose ranged from < LOQ to 0.9 g/100 g (Table 2); however, each sample group had relatively large variability in concentrations. Similar results have been shown in other studies (Escuredo et al. 2014).

### 3.2 Correlations

The Pearson correlation coefficients (*r*) between physiochemical parameters are presented in Table 3. A statistically significant (*p* < 0.05) positive correlation was observed between pH and electrical conductivity (*r* = 0.64) consistent with results reported by other researchers (Fallico et al. 2004; Kumar et al. 2018). HMF and free acidity also showed a positive correlation (*p* < 0.05, *r* = 0.50), attributed to the effect of storage conditions and aging, which influence both parameters (Bentabol Manzanares et al. 2014). While positive and negative correlations were observed among other parameters, they were either statistically nonsignificant (*p* > 0.05) or weak correlations (*r* < 0.5). Previous studies have identified significant negative correlations between HMF and diastase (Patrignani et al. 2018); however, this was not observed in our study. Some studies have found that environmental conditions such as high ambient temperature and humidity may influence HMF formation and diastase degradation in unpredictable patterns (Khan et al. 2015). Deviations can also occur due to differences in floral sources, sampling, storage, and measurement techniques (Bogdanov et al. 2004). These influences may have had an effect in the current study, especially in hot, humid regions. The maintenance of optimal conditions while the honey remains in the hive is challenging; however, postharvest practices such as minimal heating during processing and cool storage temperatures can mitigate these challenges.

**TABLE 3:** Pearson's correlation coefficients among physiochemical properties of honey.

Variables	Moisture	pH	Free acidity	Diastase	HMF	EC	WIS	Sum Fruc + Gluc	F:G	Sucrose
Moisture	**1.00**									
pH	−0.27	**1.00**								
Free acidity	0.30	−0.30	**1.00**							
Diastase	−0.24	0.07	0.09	**1.00**						
HMF	0.19	−0.13	**0.50**	−0.34	**1.00**					
EC	−0.06	**0.64**	0.33	0.01	0.12	**1.00**				
WIS	0.04	0.10	−0.04	0.04	−0.04	0.10	**1.00**			
Sum Fruc + Gluc	−0.10	−0.18	−0.17	0.17	−0.31	−0.23	0.08	**1.00**		
F:G	−0.02	0.35	−0.05	−0.08	0.06	0.33	−0.14	−0.40	**1.00**	
Sucrose	−0.14	−0.16	−0.14	−0.16	−0.08	−0.19	−0.06	−0.10	−0.07	**1.00**

*Note:* Bold numbers indicate statistically significant correlation (*p* < 0.05).

Abbreviations: EC, electrical conductivity; F:G, Fructose to Glucose ratio; HMF, hydroxymethylfurfural; Sum Fruc + Gluc, sum of fructose + glucose; WIS, water insoluble solids.

### 3.3 Principle Component and Partial Least Squares Discriminant Analysis

The chemistry data of the honey studied were processed and analyzed by chemometric methods using untargeted multivariate principal component analysis (PCA) and partial least squares‐discriminant analysis (PLS‐DA). These analyses were used to determine the chemical similarity or differences in the honey samples according to the geographical area of production.

**FIGURE 2:**
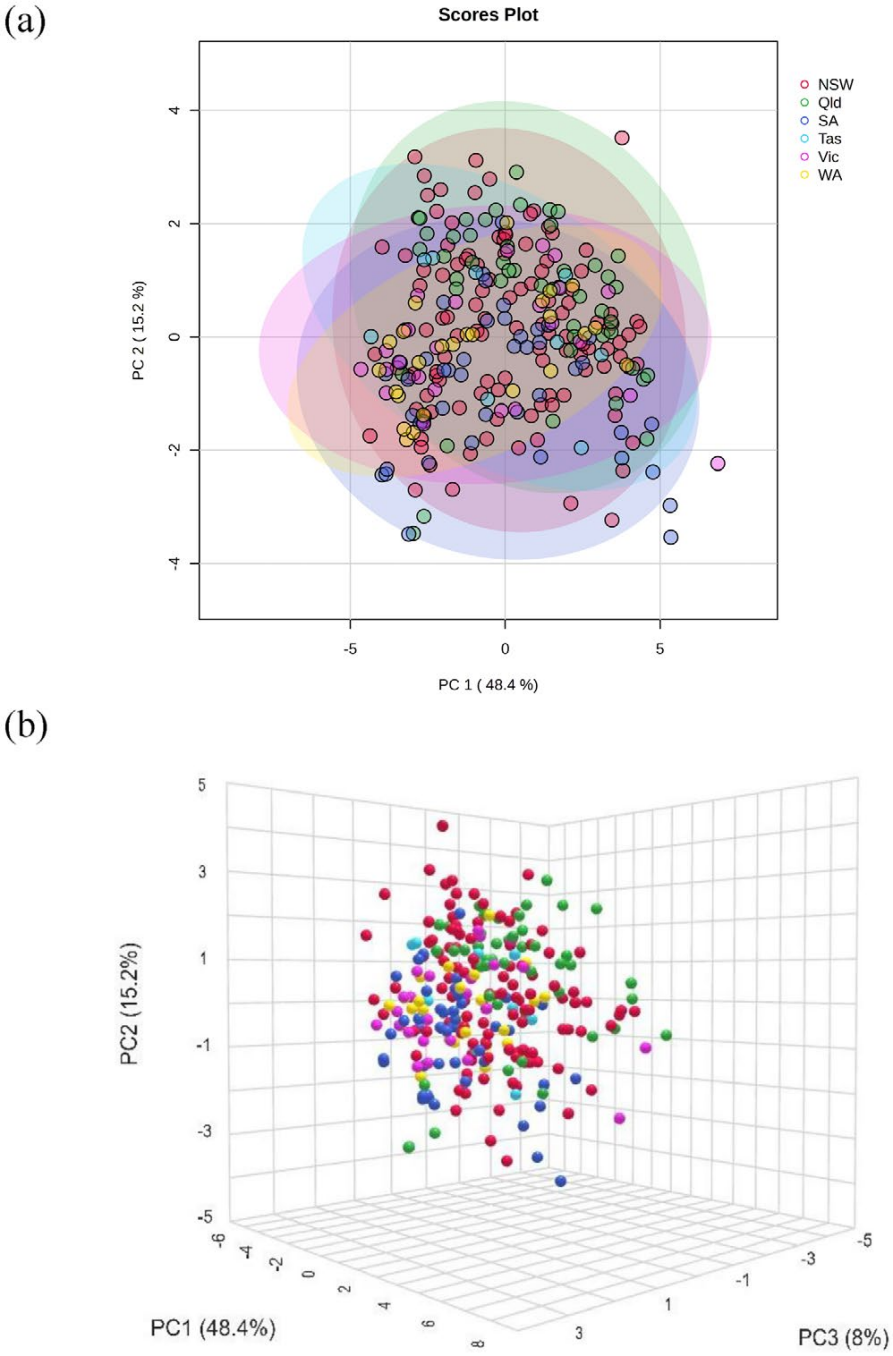
The (a) 2D and (b) 3D score plots of pricipal component analyses for Australian honey with ellipses showing 95% confidence level (red: New South Wales; green: Queensland; blue: South Australia; cyan: Tasmania; purple: Victoria, and yellow: Western Australia).

**FIGURE 3:**
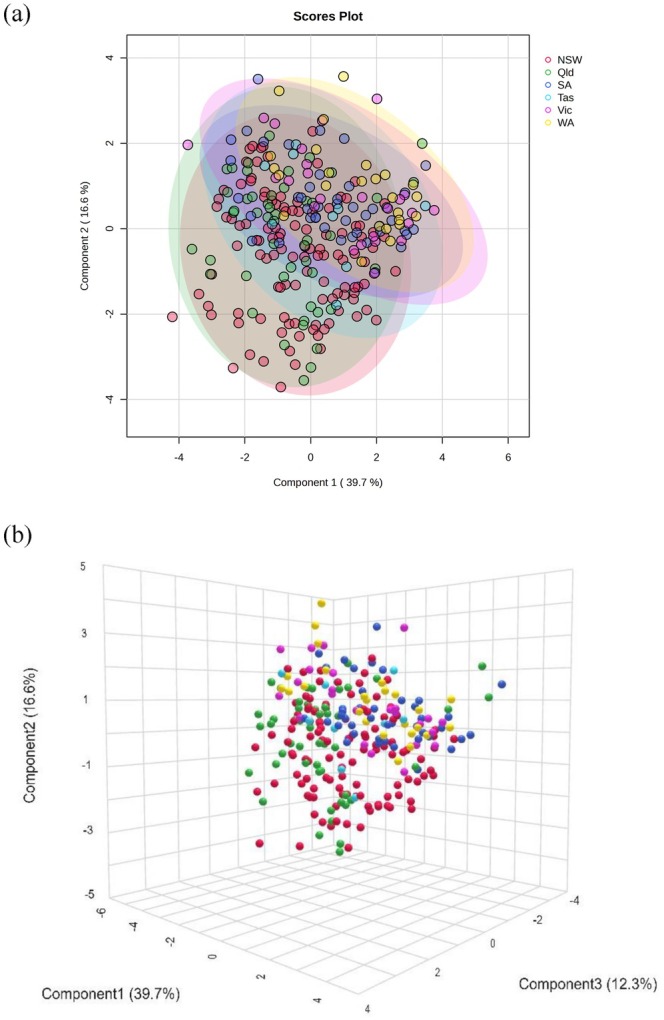
The (a) 2D and (b) 3D score plots of PLS‐DA analyses for Australian honey with ellipses showing 95% confidence level (red: New South Wales, green: Queensland, blue, South Australia; cyan, Tasmania; purple, Victoria, and yellow, Western Australia).

The PCA score plots of the data did not show any separate groupings among the states where the honey was produced (Figure 2a,b). The PLS‐DA (Figure 3a,b) showed some clustering for samples from Western Australia, South Australia, and Victoria; however, there was no distinct separation between states. Some states had a lower number of samples (Tasmania, Western Australia), which may have contributed to the lack of differentiation. Similar chemometric approaches using physio‐chemical analyses have been taken by other researchers (Karabagias et al. 2014b; Nayik and Nanda 2015; Uçurum et al. 2023). While they showed discrimination between regions, they used fewer samples or targeted floral sources. While the absence of distinct clustering in principal component analysis (PCA) is often interpreted as a limitation in the selection of variables, it is equally important to consider alternative perspectives. Such outcomes may not necessarily indicate inadequacy in the chosen variables but could reflect deeper complexities or subtleties in the data or the phenomenon under study. This could occur because of high within‐region variability, which can obscure between‐region differences, resulting in a lack of clustering. While additional parameters may have provided clearer differentiation, this was beyond the scope of this study.

## 4 Conclusions

The analysis of the chemical composition of Australian honey indicated that it is diverse. Varied climatic conditions during production and harvesting periods, combined with inconsistent flowering patterns of the honey‐producing floral sources, are likely contributing factors to this diversity. Additionally, differences in extraction, processing, and storage practices are likely contributing factors to the diversity of the end product, and these likely differed for the samples that were collected.

Australian honey generally complies with standard requirements. When a sample was outside industry standards, this could often be attributed to information provided by beekeepers, including instances of overheating during processing or undesirable storage conditions as well as climatic or geographic effects such as tropical climates. Two parameters, namely electrical conductivity (approximately 15% of samples) and water insoluble solids (approximately 25% of samples), were shown to be at elevated levels compared with industry standards. The honey analyzed in this study was raw and unfiltered, differing from the final consumer product. To assess the composition of fresh, unprocessed honey, samples were obtained directly from beekeepers. This approach preserves natural components, enabling more comprehensive analysis.

Correlations were found between some of the physiochemical parameters, including electrical conductivity and pH, while HMF and free acidity were also correlated. Insights from correlations can inform industry guidelines for storage, processing, and packaging to minimize quality degradation and meet regulatory standards.

Further investigation is warranted to fully characterize Australian honey; however, the results from this study provide a good basis for further research and give some knowledge about the diversity of honey produced. Surveying and researching honeys from regions this project did not receive samples from is also recommended. Determination of volatile compounds, phenolics, antioxidant capacity, and mineral contents have been used to enhance the understanding of the composition of honey in other countries (Karabagias et al. 2022; Nayik and Nanda 2016) and future studies into Australian honey using similar analyses could be useful. Integrating molecular and advanced spectroscopic tools such as DNA fingerprinting and infra‐red spectroscopy (FTIR) or nuclear magnetic resonance (NMR) into future studies on Australian honey could assist in differentiating honey from different regions or floral sources. This would broaden the database, help identify Australia's unique honey “fingerprint” and ensure the product's integrity is protected. This study's findings on Australian honey's chemical composition assist in informing the refinement of international standards and contribute to global efforts in combating honey adulteration, ensuring authenticity and quality across markets.

## Author Contributions

Conceptualization: J.A. Methodology: J.A., L.G., J.B. Formal analysis: J.A., L.G. Resources: J.A., L.G., J.B. Data curation: J.A., L.G. Writing – original draft preparation: J.A. Writing – review and editing: L.G., J.B. Visualization: J.A. Supervision: J.A. Project administration: J.A. Funding acquisition: J.A. All authors have read and agreed to the published version of the manuscript.

## Conflicts of Interest

The authors declare no conflicts of interest.

## Data Availability

Data are available upon request due to privacy/ethical restrictions.
